# Efficacy of Omadacycline-Containing Regimen in a Mouse Model of Pulmonary Mycobacteroides abscessus Disease

**DOI:** 10.1128/msphere.00665-22

**Published:** 2023-03-13

**Authors:** Binayak Rimal, Danielle A. Nicklas, Chandra M. Panthi, Christopher K. Lippincott, Daniel C. Belz, Elisa H. Ignatius, Daniel H. Deck, Alisa W. Serio, Gyanu Lamichhane

**Affiliations:** a Division of Infectious Diseases, Department of Medicine, School of Medicine, Johns Hopkins University, Baltimore, Maryland, USA; b Center for Nontuberculous Mycobacteria and Bronchiectasis, School of Medicine, Johns Hopkins University, Baltimore, Maryland, USA; c Division of Pulmonary and Critical Care Medicine, Department of Medicine, School of Medicine, Johns Hopkins University, Baltimore, Maryland, USA; d Division of Clinical Pharmacology, Department of Medicine, School of Medicine, Johns Hopkins University, Baltimore, Maryland, USA; e Paratek Pharmaceuticals, Inc., King of Prussia, Pennsylvania, USA; Washington University in St. Louis School of Medicine

**Keywords:** *Mycobacteroides abscessus*, omadacycline, amikacin, azithromycin, bedaquiline, biapenem, cefoxitin, clofazimine, imipenem, linezolid, rifabutin, *Mycobacterium abscessu*s

## Abstract

Mycobacteroides abscessus is an opportunistic pathogen in people with structural lung conditions such as bronchiectasis, chronic obstructive pulmonary disease, and cystic fibrosis. Pulmonary M. abscessus infection causes progressive symptomatic and functional decline as well as diminished lung function and is often incurable with existing antibiotics. We investigated the efficacy of a new tetracycline, omadacycline, in combination with existing antibiotics recommended to treat this indication, in a mouse model of M. abscessus lung disease. Amikacin, azithromycin, bedaquiline, biapenem, cefoxitin, clofazimine, imipenem, linezolid, and rifabutin were selected as companions to omadacycline. M. abscessus burden in the lungs of mice over a 4-week treatment duration was considered the endpoint. Omadacycline in combination with linezolid, imipenem, cefoxitin, biapenem, or rifabutin exhibited early bactericidal activity compared to any single drug. Using three M. abscessus isolates, we also determined the *in vitro* frequency of spontaneous resistance against omadacycline to be between 1.9 × 10^−10^ and 6.2 × 10^−10^ and the frequency of persistence against omadacycline to be between 5.3 × 10^−6^ and 1.3 × 10^−5^. Based on these findings, the combination of omadacycline and select drugs that are included in the recent treatment guidelines may exhibit improved potency to treat M. abscessus lung disease.

**IMPORTANCE** M. abscessus disease incidence is increasing in the United States. This disease is difficult to cure with existing antibiotics. In this study, we describe the efficacy of a new tetracycline antibiotic, omadacycline, in combination with an existing antibiotic to treat this disease. A mouse model of M. abscessus lung disease was used to assess the efficacies of these experimental treatment regimens. Omadacycline in combination with select existing antibiotics exhibited bactericidal activity during the early phase of treatment.

## INTRODUCTION

Mycobacteroides abscessus (also known as Mycobacterium abscessus) is a ubiquitous environmental organism which can cause opportunistic infection in susceptible individuals, including people with structural lung disease such as bronchiectasis, chronic obstructive pulmonary disease, and cystic fibrosis (1). M. abscessus disease is associated with progressive symptomatic and functional decline as well as diminished lung function (2–4). The incidence of pulmonary and extrapulmonary M. abscessus infections has been gradually rising in the United States (5, 6). Current treatment recommendations are based on repurposing existing antibiotics as there are no FDA-approved drugs for this indication (7–9), and cure rates for M. abscessus lung disease are only 30 to 50% (10, 11). One reason for this low cure rate is the intrinsic resistance of M. abscessus to most available traditional antibiotics (12, 13). Significant adverse events are frequently observed with prolonged use of many of the antibiotics that exhibit potency against M. abscessus (4). Therefore, there is an unmet need for tolerable and safe antibiotics with high efficacy against M. abscessus that can improve treatment completion and cure rates.

One of the new antibiotics being investigated for treating M. abscessus disease is omadacycline, a semisynthetic derivative of the tetracycline class (14, 15). Independent laboratories have reported its potent *in vitro* and *in vivo* activity against a diverse set of M. abscessus clinical isolates (16–21). There is emerging evidence of its utility in treating M. abscessus lung disease (22–25). However, mycobacterial diseases, including M. abscessus lung disease, require simultaneous treatment with multiple antibiotics (7–9). Therefore, any new drug with efficacy against M. abscessus will likely be combined with existing or novel antibiotics to constitute a new regimen to treat this disease.

In this study, we evaluated efficacies of dual antibiotic regimens comprised of omadacycline and other antibiotics commonly considered for treating M. abscessus disease. Amikacin, azithromycin, bedaquiline, cefoxitin, clofazimine, imipenem, and linezolid, which are included in the current recommendations to treat M. abscessus disease (7–9), were selected as companions to omadacycline, and their efficacies were evaluated *in vivo*. In addition, we also considered two antibiotics with strong preclinical data suggesting activity against M. abscessus*:* rifabutin and biapenem. There is emerging interest, based on observation of potent *in vitro* and *in vivo* activities against M. abscessus in preclinical studies, in repurposing rifabutin to treat M. abscessus disease (26, 27). Additionally, rifabutin exhibits synergism with omadacycline against a subset of M. abscessus clinical isolates (20). Biapenem (28) is a carbapenem and belongs to the same β-lactam subclass as imipenem. Although imipenem is already included as one of the candidate companions to omadacycline, we also considered biapenem as it exhibits potent activity against select M. abscessus clinical isolates that are resistant to imipenem and exhibits synergism against M. abscessus when combined with rifampicin (29, 30). Among carbapenems, only biapenem possesses permanent charge, a physiochemical property that affects pharmacokinetic properties (28). Among the antibiotics included in the recommendation, clarithromycin was not considered since azithromycin, which belongs to the same class, is included in the study and poses fewer drug-drug interaction challenges. Tigecycline was excluded as it belongs to the same chemical class as omadacycline and its long-term use is limited by frequent side effects (31). There are other antibiotics that are less frequently considered for treating M. abscessus disease, but it was beyond the scope of this study to evaluate their efficacies in combination with omadacycline against M. abscessus.

We assessed the efficacies of the above-mentioned antibiotics in combination with omadacycline against a reference strain of M. abscessus (ATCC 19977) in a mouse model of pulmonary M. abscessus disease (32). Using this model, we have evaluated efficacies of single and dual antibiotics against M. abscessus (20, 33, 34) and growth kinetics of M. abscessus in the lungs of mice (35). Appropriate single-drug and no-drug control comparators were also included. M. abscessus burden in the lungs of mice over a 4-week treatment duration was considered the endpoint. In addition, using *in vitro* approaches, we also determined the frequencies of spontaneous resistance and persistence of M. abscessus to omadacycline. In summary, we describe two vital preclinical assessments relevant to usage of omadacycline to treat M. abscessus disease.

## RESULTS

### Omadacycline in combination with linezolid, imipenem, cefoxitin, biapenem, or rifabutin exhibits early bactericidal activity.

**FIG 1:**
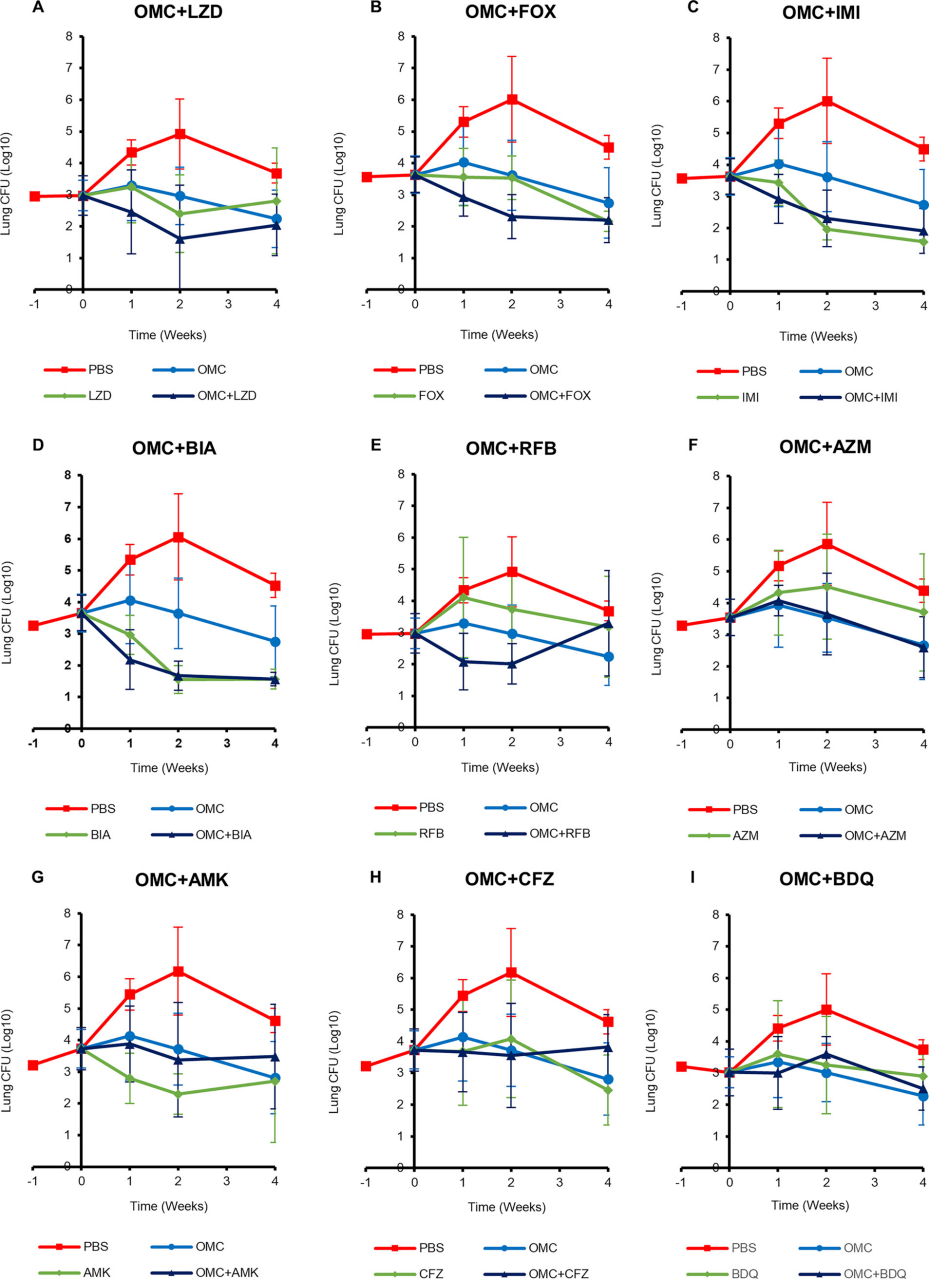
M. abscessus burdens in the lungs of mice treated with omadacycline and a companion antibiotic. Burdens of M. abscessus in the lungs of C3HeB/FeJ mice at weeks −1, 0, +1, +2, and +4 (*n *= 5 per group per time point) represented as mean CFU ± standard deviation are shown. Time point week −1 corresponds to the day after mice were infected with M. abscessus ATCC 19977, week 0 corresponds to the day antibiotic treatment was initiated, and weeks +1, +2, and +4 correspond to 1, 2, and 4 weeks, respectively, following daily treatment with stated antibiotics. PBS, phosphate-buffered saline control; OMC, omadacycline (15 mg/kg, once daily, administered via subcutaneous injection); AMK, amikacin (150 mg/kg, once daily, subcutaneous injection); AZM, azithromycin (100 mg/kg, once daily, oral); BDQ, bedaquiline (25 mg/kg, once daily, oral); BIA, biapenem (200 mg/kg per dose, twice daily, subcutaneous injection); CFZ, clofazimine (25 mg/kg, once daily, oral); FOX, cefoxitin (600 mg/kg per dose, twice daily, subcutaneous injection); IMI, imipenem (100 mg/kg per dose, twice daily, subcutaneous injection); LZD, linezolid (100 mg/kg, once daily, oral); RFB, rifabutin (20 mg/kg, once daily, oral). For efficacy assessment of dual drugs, corresponding single drugs were included at the same dose and dosing frequency. Pairwise statistical analysis between each treatment group at each time point is included in Table S1 in the supplemental material.

In the negative-control group, M. abscessus burden in the lungs increased during the first 2 weeks and stabilized thereafter with a slight decrease (Fig. 1; see statistical assessment in Table S1 in the supplemental material) as described in prior studies (32, 34). The combination of omadacycline and linezolid exhibited bactericidal activity against M. abscessus lung infection from the onset until the end of the first 2 weeks of treatment (Fig. 1A). The mean lung M. abscessus burdens in mice treated with the omadacycline-plus-linezolid combination were lower than those in mice that received either drug alone. When administered alone, omadacycline and linezolid exhibited bacteriostatic activity during this same period. However, after 2 weeks of treatment, M. abscessus burden in mice that received this combination increased slightly, which mirrored the increase in mice that received linezolid only.

10.1128/msphere.00665-22.1 TABLE S1 Statistical assessment of lung M. abscessus burdens between groups of mice receiving different treatments. *P* values were determined from a single-factor analysis of variance (ANOVA) of M. abscessus burdens in the lungs of mice in different treatment groups at 1-, 2-, and 4-week time points following treatment as shown in Fig. 1. Column 1: the panels in Fig. 1 to which the data shown in the row correspond. Column 2: comparison between treatment groups—phosphate-buffered-saline (PBS), omadacycline (OMC), linezolid (LZD), cefoxitin (FOX), imipenem (IMI), biapenem (BIA), azithromycin (AZM), rifabutin (RFB), amikacin (AMK), clofazimine (CFZ), and bedaquiline (BDQ) and drug combinations. * represents a *P* value of ≤0.05, and ** represents a *P* value of ≤0.01. Download Table S1, PDF file, 0.1 MB. Copyright © 2023 Rimal et al. 2023 Rimal et al. https://creativecommons.org/licenses/by/4.0/ This content is distributed under the terms of the Creative Commons Attribution 4.0 International license.

The combinations of omadacycline with each of the three β-lactams evaluated here, cefoxitin, imipenem, and biapenem, exhibited similar overall bactericidal activities against M. abscessus disease. From the onset of treatment until 2 weeks, the combination omadacycline-plus-cefoxitin exhibited bactericidal activity whereas cefoxitin alone was bacteriostatic (Fig. 1B). After 2 weeks, cefoxitin alone also exhibited bactericidal activity. The combination omadacycline-plus-imipenem exhibited bactericidal activity throughout the treatment period (Fig. 1C). The efficacy of the omadacycline-plus-biapenem combination (Fig. 1D) was similar to that of omadacycline-plus-imipenem. Although statistically not significant, the combination of omadacycline-plus-biapenem produced a slightly greater reduction in lung M. abscessus burden than did omadacycline-plus-imipenem. Cefoxitin and imipenem are already included in the current recommendation to treat M. abscessus disease (7, 8); other β-lactams such as doripenem, tebipenem, and biapenem exhibit therapeutically useful activity against M. abscessus (30), and select dual β-lactam combinations exhibit synergy against M. abscessus (36, 37). The combination of omadacycline-plus-rifabutin exhibited bactericidal activity during the first 2 weeks of treatment (Fig. 1E). However, at the end of 4 weeks of treatment, lung M. abscessus burden in this group of mice increased and was higher than that in mice that received omadacycline alone and comparable to that in mice that received rifabutin or phosphate-buffered saline (PBS) only.

### Efficacy of omadacycline in combination with azithromycin, amikacin, clofazimine, or bedaquiline.

M. abscessus lung burdens in mice that received omadacycline alone or the omadacycline-plus-azithromycin combination were indistinguishable throughout the study duration (Fig. 1F). Therefore, efficacy of omadacycline against M. abscessus lung infection was unaltered when combined with azithromycin. In mice that received the combination of omadacycline-plus-amikacin (Fig. 1G) or omadacycline-plus-clofazimine (Fig. 1H), mean lung M. abscessus burdens at the conclusion of treatment were higher than those in mice that received either omadacycline, amikacin, or clofazimine alone. Although these differences were not statistically significant (supplemental material), this evidence suggests that efficacies of the combinations of omadacycline-plus-amikacin and of omadacycline plus clofazimine may not be superior to the efficacies of individual drugs against M. abscessus infection. Similarly, the mean lung M. abscessus burden in mice that received the combination of omadacycline-plus-bedaquiline was slightly higher than that in mice that received omadacycline only (Fig. 1I). Therefore, the addition of bedaquiline did not improve the efficacy of omadacycline against M. abscessus lung infection.

### Mutant selection window (MSW) and mutant prevention concentration (MPC) of omadacycline.

Three M. abscessus isolates, ATCC 19977 and recent clinical isolates M9501 and M9507 obtained from the Johns Hopkins Hospital Clinical Microbiology Laboratory, were included. These isolates were selected based on their MIC profile to a panel of antibiotics in cation-adjusted Mueller-Hinton broth (CAMHB) and Middlebrook 7H9 broth, published in 2022 (20). The MICs of drugs commonly used to treat M. abscessus disease against M9501 are comparable to MICs against ATCC 19977 except for the MIC of clarithromycin (1 μg/mL and ≤0.06 μg/mL against ATCC 19977 and M9501, respectively). The MICs of amikacin, clarithromycin, and azithromycin against M9507 are much higher (>256 μg/mL, 4 μg/mL, and 32 μg/mL, respectively).

MSW represents the lowest and highest omadacycline concentrations at which distinct M. abscessus colonies could be isolated. MPC represents the omadacycline concentration at or above which resistant M. abscessus could not be isolated. The MSW ranged from 3.75 to 7 μg/mL (Table 1). Omadacycline MPCs for ATCC 19977 and M9507 were 7.5 μg/mL and 7.0 μg/mL, respectively. An MPC was not determined for isolate M9501.

**TABLE 1:** Omadacycline MIC, MSW, MPC, Freq^r^, and Freq^p^ of M. abscessus isolates^*a*^

Strain	MIC (μg/mL)	MSW (μg/mL)	MPC (μg/mL)	Freq^r^ at day 7	Freq^r^ post-day 7	Freq^p^ post-day 7
ATCC 19977	0.375	3.75–7.5	7.5	≤1.93 × 10^−10^	1.93 × 10^−10^	5.28 × 10^−6^
M9501	0.25	≥6	ND	≤5.31 × 10^−10^	2.66 × 10^−6^	5.31 × 10^−6^
M9507	0.5	5–7	7	≤6.20 × 10^−10^	1.61 × 10^−6^	1.29 × 10^−5^

a Mutant selection window (MSW) represents the lowest and highest omadacycline concentrations at which distinct M. abscessus colonies could be isolated. Mutant prevention concentration (MPC) represents the omadacycline concentration at or above which resistant M. abscessus could not be isolated. Freq^r^ represents the frequency of selection of spontaneous mutants of M. abscessus that are resistant to omadacycline. Freq^p^ represents the frequency of subpopulations of M. abscessus that are transiently tolerant and can persist in the presence of omadacycline. M. abscessus CFU enumerated at 7 days of exposure to omadacycline and used to determine Freq^r^ is listed under “Freq^r^ at day 7.” Any additional CFU that appeared after 7 days of incubation on omadacycline-containing medium and exhibited an elevated omadacycline MIC was used to determine the frequency of resistant mutants and listed under “Freq^r^ post-day 7.” Similarly, additional CFU that appeared after 7 days but did not exhibit an elevated omadacycline MIC were defined as persisters, and their frequencies are listed under “Freq^p^ post-day 7.” ND, not determined.

### Frequency of resistance and persistence to omadacycline.

All tested M. abscessus isolates showed no growth on omadacycline-containing CAMH agar plates at the standard day 7 time point (38). To avoid underestimation of frequency of resistant mutants, if no colonies were observed, CFU counts were rounded to 1 CFU to determine an equivalent standard of frequency of spontaneous resistance (Freq^r^) to omadacycline at the day 7 time point. As such, resistance frequencies of ATCC 19977 as well as M9501 and M9507 are ≤1.93 × 10^−10^, ≤5.31 × 10^−10^, and ≤6.20 × 10^−10^, respectively, at the standard day 7 time point (Table 1).

M. abscessus CFU was again enumerated after additional duration of incubation to detect any slow-growing resistant mutants or persister subpopulations. We were able to observe new CFU only up to an additional 7 days of incubation. At this time point, designated the “post-day 7” time point, total CFU was enumerated, and nine randomly sampled colonies derived from each isolate were selected and omadacycline MIC was determined.

For ATCC 19977, none of the putative mutants (0 of 9) showed an omadacycline MIC shift after subsequent testing to suggest resistance. For isolate M9501, three putative mutants (3 of 9) had increased omadacycline MICs, one with a 10.7-fold increase (postexposure MIC of 4 μg/mL) and two mutants with a 5.3-fold increase (postexposure MIC of 2 μg/mL) compared to the parent MIC of 0.25 μg/mL (Table S2). For isolate M9507, one putative mutant (1 of 9) had a 10-fold increase (postexposure MIC of 5 μg/mL) compared to the parent MIC of 0.5 μg/mL. All remaining putative mutants with MIC shifts of <4-fold were considered to be persisters as they survived exposure to omadacycline through transient nonheritable adaptations.

10.1128/msphere.00665-22.2 TABLE S2 M. abscessus isolates selected for generational passaging with omadacycline MIC shifts at T0 and T5 generations. T0 represents colonies isolated after exposure to omadacycline. T5 represents colonies derived from T0 isolates after five rounds of passage in drug-free CAMH broth and agar. The first column lists two clinical isolates whose derivates after exposure to omadacycline for isolation of resistant mutants consistently exhibited omadacycline MICs elevated 4-fold (normalized to omadacycline MIC against the parental strain), defining resistance. The second column is omadacycline MICs for isolates listed in column 1. The third column lists the isolates derived from the parent strain in column 1 after exposure to omadacycline and confirmed to exhibit a stable increase in omadacycline MIC. This is the T0 generation. In column 4, omadacycline MICs of T0 generation mutants are listed. In column 5, the fold change in omadacycline MIC of T0 mutants compared to that for parent strains is listed (calculated as column 4 − column 2). Parent strains (listed in column 1) and T0 mutants were passaged for five rounds in drug-free broth and agar, and the omadacycline MIC for each resulting isolate was determined. Omadacycline MICs of parent strains after passage in drug-free medium are listed in column 6. Similarly, omadacycline MICs for T0 mutants after passage in drug-free medium are listed in column 7. The fold changes for omadacycline MICs in T5 mutants compared to those for T0 are listed in column 8. Download Table S2, PDF file, 0.07 MB. Copyright © 2023 Rimal et al. 2023 Rimal et al. https://creativecommons.org/licenses/by/4.0/ This content is distributed under the terms of the Creative Commons Attribution 4.0 International license.

Using the proportions of persistent and resistant isolates for each M. abscessus parent strain, total CFU enumerated after day 7 was classified into colonies with omadacycline persistence (OMC^p^) and resistance (OMC^r^). For ATCC 19977, no CFU counts were obtained, and so the OMC^r^ CFU counts were rounded to 1 CFU to avoid underestimation and enable calculations of CFU post-day 7. As such, resistance frequencies of ATCC 19977, M9501, and M9507 are 1.93 × 10^−10^, 2.66 × 10^−6^, and 1.61 × 10^−6^, respectively, at the post-day 7 time point (Table 1). While ATCC 19977 maintained a low frequency of resistance (1.93 × 10^−10^) after 7 days of omadacycline exposure, the frequency of resistance of isolates M9501 and M9507 increased to 2.66 × 10^−6^ and 1.61 × 10^−6^, respectively, due to the appearance of additional OMC^r^ CFU. The frequencies of persister subpopulations present in cultures of ATCC 19977, M9501, and M9507 were 5.28 × 10^−6^, 5.31 × 10^−6^, and 1.29 × 10^−5^, respectively.

The four mutants (M9501-T52, M9501-T56, M9501-T57, and M9507-T51) with omadacycline MIC values >4-fold that of the parent were selected for sequential generational passaging in a drug-free environment for five rounds to determine if the elevated omadacycline MIC values were the result of genetically stable omadacycline resistance mutations. After five rounds of passage in drug-free broth, the omadacycline MIC for the M9501 parent strain was 0.75 μg/mL, while the MICs against mutants M9501-T52, M9501-T56, and M9501-T57 were 3 μg/mL, 2 μg/mL, and 1.5 μg/mL, respectively, representing 4-fold, 2.7-fold, and 2-fold increases in MIC, respectively. These data demonstrate that the initial MIC shifts (5.3- to 10.7-fold increases in MIC) observed at day 0 were transient and not heritable. Further, the MICs observed after five passages for the mutants compared to the parent are all within the standard variability of a MIC assay (2- to 4-fold change).

For the M9507 parent strain, the omadacycline MIC after five rounds of passage in drug-free broth was 0.75 μg/mL, while the MIC value against the mutant (M9507-T1) was 6 μg/mL. This represents an 8-fold increase in MIC, indicating the possibility of stable resistance in this mutant, although the fold increase in MIC was reduced from 10-fold at day 0 to 8-fold after five rounds of passage.

### Exposure to omadacycline does not promote cross-resistance to other antibiotics.

After five rounds of passage of putative mutants and their parent strains M9501 and M9507 in drug-free medium, MICs against an eight-drug panel (omadacycline, azithromycin, cefoxitin, tigecycline, clarithromycin, cefdinir, imipenem, and rifabutin) were determined, to assess for a stably elevated omadacycline MIC and for any cross-resistance to comparator drugs (Table S3). MIC assays were performed in duplicate, and the average MIC was calculated. The MICs of comparator drugs were unchanged for the mutants tested, specifically, azithromycin, cefoxitin, tigecycline, clarithromycin, cefdinir, imipenem, or rifabutin. These data demonstrate that susceptibility of M. abscessus to drugs of classes similar to and different from that of omadacycline is not negatively impacted.

10.1128/msphere.00665-22.3 TABLE S3 MIC (micrograms per milliliter) of antibiotics commonly used to treat M. abscessus disease panel for the fifth-generation (T5) mutants and respective antibiotic MIC shifts. (Top panel) MICs (micrograms per milliliter) of omadacycline (OMC), azithromycin (AZM), cefoxitin (FOX), tigecycline (TGC), clarithromycin (CLR), cefdinir (CDR), imipenem (IMI), and rifabutin (RFB) against parent strain M9501 and mutant derivatives after exposure to omadacycline are shown. Column 1: list of antibiotics. Column 2: MIC (micrograms per milliliter) of antibiotics listed in column 1 for strain M9501. Column 3: MIC of antibiotics for strain M9501 after five rounds of passage in drug-free medium. Columns 4, 6, and 8: MIC of antibiotics for M9501-T52, -T56, and -T57 (the three mutants with consistently elevated omadacycline MICs). Columns 5, 7, and 9: fold change in MICs of antibiotics listed in column 1 for mutants compared to MICs of drugs versus parent strain M9501-T5 in column 3. (Bottom panel) MIC (micrograms per milliliter) of antibiotics against parent strain M9507 and mutant derivate M9507-T51 after exposure to omadacycline. Column 1: list of antibiotics. Column 2: MIC (micrograms per milliliter) of antibiotics listed in column 1 for strain M9507. Column 3: MIC of antibiotics for strain M9507 after five rounds of passage in drug-free medium. Column 4: MIC of antibiotics for M9507-T51. Column 5: fold change in MICs of antibiotics listed in column 1 for mutants compared to MICs of drugs versus parent strain M9507-T5 in column 3. Download Table S3, PDF file, 0.1 MB. Copyright © 2023 Rimal et al. 2023 Rimal et al. https://creativecommons.org/licenses/by/4.0/ This content is distributed under the terms of the Creative Commons Attribution 4.0 International license.

## DISCUSSION

M. abscessus lung disease has been historically treated with a regimen of multiple drugs to improve overall treatment efficacy and to minimize selection of spontaneous resistant mutants (7–9, 39). Therefore, any new drug with activity against M. abscessus is likely to be considered only as a component of a multidrug regimen. Four independent studies have reported potent *in vitro* activity of omadacycline against M. abscessus as demonstrated by MIC_90_ values ranging from 0.5 to 2 μg/mL against a diverse set of recent clinical isolates (17–20). In a mouse model of pulmonary M. abscessus disease, once-daily administration of 15 mg of omadacycline per kg of body weight, equivalent to a 300-mg standard oral dose in humans, resulted in a 1- to 3-log_10_ reduction in M. abscessus burden in the lungs at the end of a 4-week treatment period against four independent isolates causing infection, including three recent clinical isolates (20). This same study also demonstrated that MICs of omadacycline against M. abscessus isolates causing infection did not alter following continuous exposure to this drug in mice for 4 weeks.

To develop a new regimen to treat M. abscessus disease, a new drug is likely to be combined with one or more drugs that are already included in the treatment guidelines (7–9). Ideally, the combination of a new drug and existing drugs will exhibit synergism in efficacy against M. abscessus. Omadacycline exhibits synergism *in vitro* with clarithromycin, azithromycin, cefdinir, linezolid, and rifabutin against a subset of M. abscessus isolates and does not exhibit antagonism with any of the antibiotics currently recommended to treat M. abscessus disease (20, 40). In this study, we evaluated the efficacy of omadacycline in combination with a subset of these drugs as well as imipenem, cefoxitin, clofazimine, and amikacin, as they are included in the current recommendations to treat M. abscessus lung disease. We also included rifabutin and biapenem for the above-mentioned reasons.

The combination of omadacycline and linezolid exhibited bactericidal activity from the onset of treatment up to 2 weeks and bacteriostatic activity thereafter. Overall, the mean M. abscessus burden in mice treated with this combination was lower than that in mice treated with either drug alone, suggesting enhanced potency of omadacycline and linezolid in combination. Both drugs bind to the bacterial 70S ribosomal complex and inhibit protein synthesis. More specifically, omadacycline directly targets the 30S subunit while linezolid targets the 50S subunit in the ribosome complex (14, 41, 42). It will require further study to determine whether binding to two distinct sites in the 70S ribosome complex by omadacycline and linezolid inhibits ribosome function more effectively than binding by either drug alone, a possible explanation for the enhanced efficacy of this combination against M. abscessus.

The combinations of omadacycline and each of the three β-lactams evaluated here, cefoxitin, imipenem, and biapenem, exhibited similar overall efficacies against M. abscessus disease characterized by potent bactericidal activity. During the first 1 or 2 weeks of treatment, omadacycline in combination with the β-lactams reduced lung M. abscessus burden more rapidly than either drug alone. But, at the end of 4 weeks of treatment, each individual β-lactam reduced lung M. abscessus burden to the same extent as the combination. β-Lactams inhibit synthesis and metabolism of peptidoglycan, an essential component of the bacterial cell wall (43). Two β-lactams, cefoxitin and imipenem, are already included in the current recommendation to treat M. abscessus disease (7, 8, 44); several others, such as doripenem, tebipenem, and biapenem, exhibit therapeutically useful activity against M. abscessus (29, 45), and select dual β-lactam combinations exhibit synergy against M. abscessus (33, 36, 37, 46). As the combination of omadacycline and β-lactams exhibits enhanced potency during the first weeks of treatment (Fig. 1B to D), we hypothesize that simultaneous inhibition of ribosome function and cell wall peptidoglycan biosynthesis kills M. abscessus more effectively than inhibition of either of these essential pathways achieved by each class of drug alone.

Combination therapy has been demonstrated to reduce the frequency of selection of spontaneous resistant mutants (47, 48). Therefore, the combination of omadacycline and another drug effective against M. abscessus, such as a β-lactam and linezolid, may also reduce the frequency of selection of spontaneous resistant mutants. Our results demonstrate that not only was it difficult to select M. abscessus mutants on omadacycline-containing plates that had resulting omadacycline MIC values of >4-fold that of the parent strain, but also that for all but one of the putative mutants the observed elevated omadacycline MIC was transient and not stable after passaging, suggesting that there were no heritable alterations conferring omadacycline resistance. The 10^−5^ to 10^−6^ frequency of M. abscessus persisters to omadacycline may not be surprising as the drug exerts a bacteriostatic effect on this organism. We hypothesize that the combination of omadacycline and a bactericidal antibiotic such as a β-lactam may exhibit very low frequencies of spontaneous resistance and persistence. Testing this hypothesis was beyond the scope of this study; however, it was encouraging that there was no evidence of relapsing infection even in mice that received only a single drug. The M. abscessus burden of 3 to 4 log_10_ at the time of treatment initiation is likely insufficient for random mutations to accumulate in the sites targeted by these drugs and to afford resistance. Lung M. abscessus burden can reach 6 log_10_ (Fig. 1) after 2 or 3 weeks of dexamethasone administration and as high as 7 to 8 log_10_ if higher doses of dexamethasone are used (20). This study demonstrated that unpredictable numbers of mice die at this M. abscessus burden. Therefore, such high burdens may not constitute a reliable and reproducible experimental design to conclude whether combinations of drugs with enhanced efficacy also reduce the frequency of spontaneous resistant mutant selection.

Two attributes sought after in regimens to treat mycobacterial infections are early bactericidal activity and sterilizing activity (49). This study demonstrates that omadacycline in combination with linezolid, imipenem, cefoxitin, biapenem, or rifabutin exhibits potent early bactericidal activity against M. abscessus lung infection in C3HeB/FeJ mice. Such bactericidal activity would be highly beneficial to patients with M. abscessus disease. Also, combination therapy has been demonstrated to reduce the frequency of selection of spontaneous resistant mutants (47). Therefore, the combination of omadacycline and another drug effective against M. abscessus, such as a β-lactam, linezolid, or rifabutin, may also reduce the frequency of resistant mutant selection.

These data are timely as the efficacy and safety of omadacycline (300-mg oral dose) to treat M. abscessus lung disease in humans are currently being studied in a phase 2, double-blind, randomized clinical trial (ClinicalTrials.gov identifier NCT04922554). This study demonstrates several findings with important implications that should be further explored through human clinical trials. Reassuringly, this study further supports prior data suggesting that omadacycline MICs are robust and that emergent resistance has not been seen in mouse models even with monotherapy (20). Current clinical guidelines for treatment of M. abscessus lung disease recommend at least three active drugs based on *in vitro* susceptibilities, though only azithromycin and amikacin sensitivities are validated for interpretation (7–9, 50). These data suggest that designing treatment regimens where individual drugs are considered without respect to companion drugs may compromise treatment efficacy. Additionally, it is encouraging that imipenem and cefoxitin improved bacterial killing and both are readily available for clinical use. While the combination of linezolid and omadacycline did improve efficacy compared to monotherapy with either drug, it did not demonstrate sustained bactericidal activity. Unfortunately, use of linezolid is often limited clinically by duration-dependent myelosuppression (51, 52). However, if confirmed in future studies, these data suggest there may still be a niche role for linezolid. For example, a short duration of treatment in combination with omadacycline (2 to 4 weeks) could prove beneficial, including in scenarios where timely outpatient treatment initiation is necessary but parenteral antibiotic therapy (i.e., imipenem or cefoxitin) cannot be coordinated urgently. Azithromycin coadministration with omadacycline demonstrated some additive benefit without detrimental effect, which is critical since macrolides are recommended for treatment of M. abscessus lung disease due to immunomodulatory properties regardless of baseline resistance (50). The deleterious impact on bacterial burden seen with coadministration of omadacycline with either clofazimine or amikacin is important and warrants further exploration in addition to exploring whether similar findings are seen with coadministration of omadacycline and amikacin liposomal inhaled suspension (ALIS).

Omadacycline is a promising oral agent for treatment of M. abscessus lung disease. This study demonstrates that activity of omadacycline against M. abscessus can be further improved with companion drugs commonly used for treatment of M. abscessus lung disease and that selection of antibiotic regimens without consideration of these combined effects may compromise treatment efficacy. The results from this study support further evaluation of omadacycline in combination with two or more drugs.

## MATERIALS AND METHODS

### Bacterial strains, culture media, and *in vitro* growth conditions.

M. abscessus strain ATCC 19977 (53), commonly used as a reference isolate, was obtained from the ATCC (Manassas, VA) and authenticated by genome sequencing (46). It was grown in Middlebrook 7H9 broth (Difco; 271310) supplemented with 10% albumin-dextrose-saline (ADS) enrichment, 0.5% glycerol, and 0.05% Tween 80 as described previously (54) with constant shaking in an orbital shaker at 220 rpm, 37°C. M. abscessus from mouse lungs was recovered by inoculating appropriate 10-fold dilutions of lung homogenates on Middlebrook 7H11 selective agar (Difco; 283810) supplemented with 10% ADS, 0.5% glycerol, 50 mg/L cycloheximide (Sigma-Aldrich; C7698), and 50 mg/L carbenicillin (Fisher; 50-213-247) and incubating at 37°C for 5 days. For determination of spontaneous resistance and persistence to omadacycline, M. abscessus recent clinical isolates M9501 and M9507, obtained from the Johns Hopkins Hospital Clinical Microbiology Laboratory, were included in addition to ATCC 19977. These isolates were genotyped by whole-genome sequencing (46).

### Antibiotics.

Omadacycline was provided by Paratek Pharmaceuticals in powder form. Each 100-mg vial was dissolved in sterile 1× phosphate-buffered saline (PBS), pH 7.4 (Quality Biologicals; 114-058-101), to prepare a 3.75-mg/mL solution. Amounts sufficient for each administration were aliquoted in 5-mL polypropylene tubes and stored at −20°C. Each aliquot was thawed immediately prior to use. Aliquots necessary for each week were prepared at the beginning of the treatment week. Clofazimine was procured from Sigma-Aldrich (C8895). Pharmaceutical-grade amikacin (CAS no. 39831-55-5), azithromycin (CAS no. 8390501-5), bedaquiline fumarate (CAS no. 845533-86-0), biapenem (CAS no. 120410-24-4), cefoxitin sodium (CAS no. 33564-30-6), imipenem (CAS no. 74431-23-5), linezolid (CAS no. 165800-03-3), and rifabutin (CAS no. 72559-06-9), in powder form, were procured from Octagon Chemicals Ltd. Necessary amounts of amikacin, azithromycin, bedaquiline fumarate, biapenem, cefoxitin, imipenem, linezolid, and rifabutin for each dose were weighed at the beginning of the corresponding efficacy study and stored in 5- or 25-mL sterile polypropylene tubes at −20°C. An 0.05% agarose solution was prepared by adding 50 mg Bacto agar (BD; 214010) to 100 mL 1× PBS, pH 7.4, and autoclaving for 10 min at 121°C. An 0.5% carboxymethyl cellulose solution was prepared by adding 500 mg of this powder (Sigma-Aldrich; C5013) in 100 mL 1× PBS, pH 7.4, dissolving by stirring for 4 to 5 h, and autoclaving for 10 min at 121°C. A 20% 2-hydroxypropyl-β-cyclodextrin (HPCD) (Sigma-Aldrich; 332593) solution was prepared as described previously (55). Briefly, 20 g of HPCD powder was transferred to a 100-mL borosilicate bottle, and 75 mL of sterile deionized (DI) water was added and stirred with a magnetic stirrer until a clear solution was obtained (~30 min). A 1.5-mL volume of 1 N HCl was added, and the final volume was brought to 100 mL by adding sterile DI water. This solution was filtered through a 0.22-μm acetate cellulose filter and stored at +4°C until use.

For amikacin, prior to administration, each aliquot was dissolved in 1× PBS, pH 7.4, by sonicating for 10 s to prepare a 37.5-mg/mL solution. All sonications were performed using the Sonic Dismembrator (model 100; Fisher Scientific) set at 50% power. For azithromycin, prior to administration, each aliquot was dissolved in 1× PBS, pH 7.4, by sonicating for 10 s to prepare a 100-mg/mL solution. For bedaquiline fumarate, the amount necessary for 1 week of administration was weighed into a 100-mL borosilicate bottle. A precise volume of 20% HPCD solution was added to obtain 3.125 mg/mL bedaquiline and dissolved by stirring for 2 to 3 h. For biapenem, prior to administration, each aliquot was dissolved in 1× PBS, pH 7.4, by sonicating for 10 s to prepare a 25-mg/mL solution. For cefoxitin, prior to administration, each aliquot was dissolved in 1× PBS, pH 7.4, by vortexing for 30 s to prepare a 75-mg/mL solution. For clofazimine, prior to administration, 0.05% agarose was added to prepare a 3.125-mg/mL suspension. This mixture was vortexed at high speed for 5 min. For imipenem, prior to administration, each aliquot was dissolved in 1× PBS, pH 7.4, by sonicating for 10 s to prepare a 12.5-mg/mL solution. For linezolid, prior to administration, 0.5% carboxymethyl cellulose was added to prepare a 12.5-mg/mL suspension. This mixture was vortexed at high speed for 2 min. For rifabutin, prior to administration, 0.05% agarose (Bacto agar; BD; 214010) was added to prepare a 2.5-mg/mL suspension. This mixture was vortexed at high speed for 5 min.

Dexamethasone powder was procured from Sigma-Aldrich (catalog no. D1756). The amount necessary for each dosage was weighed and stored in 25-mL polypropylene tube. Forty-two aliquots (one dose per day × 42 days) were prepared at the beginning of the study and stored at −20°C. Prior to administration, 1× PBS, pH 7.4, was added to prepare a 1.25-mg/mL solution and vortexed at high speed for 2 min to prepare a solution. This preparation appears cloudy white.

### Mice, infection, and efficacy studies.

C3HeB/FeJ mice, female, 5 to 6 weeks old, were procured from Jackson Laboratories (Bar Harbor, ME). Mice were treated with 5-mg/kg once-daily dexamethasone beginning 1 week prior to infection with M. abscessus and throughout the duration of the study as described in the protocol for a mouse model of pulmonary M. abscessus infection (32). For this, 100 μL of a 1.25-mg/mL solution of dexamethasone was injected into each mouse, daily, 7 days a week, throughout the experiment duration. M. abscessus grown to logarithmic phase (optical density at 600 nm [OD_600_] of ~0.95 to 1.2) was used to prepare a 10-mL suspension at an OD_600_ of 0.1 by diluting in Middlebrook 7H9 broth. Mice were infected using the Inhalation Exposure System (Glas-Col, Terre Haute, IN) with an aerosol of this suspension. The infection cycle included preheating for 15 min, aerosol nebulization for 30 min, and cloud decay for 30 min followed by decontamination for 15 min. This study was conducted in five phases, with two two-drug combinations and an appropriate single-drug control per phase. The exact protocol was repeated in each phase to enable comparison of data.

Five mice per time point per treatment group were allocated. Treatment with antibiotics was initiated 1 week postinfection. Amikacin, 150 mg/kg, once daily, was administered by subcutaneous injection of an 0.1-mL bolus of a 37.5-mg/mL solution in the hind dorsal flank. Azithromycin, 100 mg/kg, once daily, was administered by oral gavage of an 0.2-mL bolus of a 12.5-mg/mL solution. Bedaquiline, 25 mg/kg, once daily, was administered by oral gavage of an 0.2-mL bolus of a 3.125-mg/mL solution. Biapenem, 200 mg/kg per dose, twice daily, was administered by subcutaneous injection of an 0.2-mL bolus of a 25-mg/mL solution in the hind dorsal flank. Cefoxitin, 600 mg/kg per dose, twice daily, was administered by subcutaneous injection of an 0.2-mL bolus of a 75-mg/mL solution in the hind dorsal flank. Clofazimine, 25 mg/kg, once daily, was administered by oral gavage of an 0.2-mL bolus of a 3.125-mg/mL suspension. Imipenem, 100 mg/kg per dose, twice daily, was administered by subcutaneous injection of an 0.2-mL bolus of a 12.5-mg/mL solution in the hind dorsal flank. Linezolid, 100 mg/kg, once daily, was administered by oral gavage of an 0.2-mL bolus of a 12.5-mg/mL solution. Omadacycline, 15 mg/kg, once daily, was administered by subcutaneous injection of an 0.1-mL bolus of a 3.75-mg/mL solution in the hind dorsal flank. As omadacycline lacks oral bioavailability in mice, a subcutaneous dose that is equivalent to a 300-mg oral dose in humans was used as determined in a prior study (20). Rifabutin, 20 mg/kg, once daily, was administered by oral gavage of an 0.2-mL bolus of a 2.5-mg/mL suspension. To administer 0.1-mL bolus injections, an 0.5-mL syringe with a 27-gauge needle (BD; 305620) was used. To administer 0.2-mL bolus injections, a 1-mL syringe with a 26-gauge needle (BD; 309597) was used. To administer an 0.2-mL oral bolus, a 22-gauge curved gavage needle, with a 2-mm tip diameter (Gavageneedle.com; AFN2425C), and a 1-mL slip-tip syringe (BD; 309659) were used.

For determination of lung M. abscessus CFU burden, time points assessed included 24 h postinfection (designated week −1), on the day of treatment initiation (week 0), and at the completion of 1, 2, and 4 weeks of treatment (weeks +1, +2, and +4, respectively). Mice were sacrificed, and lungs were obtained and homogenized with 0.2-mm glass beads in a mechanical homogenizer (Minilys; Bertin Technologies) at 5,000 rpm for 0.5 min. Undiluted lung homogenates and 10-fold dilutions prepared in 1× PBS, pH 7.4, were inoculated onto Middlebrook 7H11 selective agar and incubated at 37°C for 5 days, and CFU was enumerated.

### Ethics.

Animal procedures used in the studies described here were performed in adherence to the Johns Hopkins University Animal Care and Use Committee and to the national guidelines.

### Data analysis.

CFU data were analyzed to determine mean ± standard deviation at each time point in each treatment group and graphed using GraphPad Prism. Statistical comparisons of M. abscessus CFU at each time point between mouse groups that received PBS or companion drug (linezolid, cefoxitin, imipenem, biapenem, azithromycin, rifabutin, amikacin, clofazimine, or bedaquiline) versus groups that received omadacycline in combination with above-mentioned antibiotics as companion drugs were assessed using a single-factor analysis of variance (ANOVA) (see Table S1 in the supplemental material). Significance was determined at 95% confidence intervals. A *P* value of <0.05 was considered significant (* represents *P* ≤ 0.05; ** represents *P* ≤ 0.01). A *P* value of >0.05 was considered not significant and was represented as NS.

ANOVA tests were used to determine whether there is an enhanced activity of omadacycline in combination with any of the above-mentioned antibiotics, thus indicating the improved efficacy when combined with omadacycline. At the end of time points week +1, week +2, and week +4, the mean lung M. abscessus burdens in mice treated with omadacycline in combination with linezolid, imipenem, biapenem, azithromycin, or bedaquiline were statistically significant compared to the mice treated with PBS alone but not significant compared to the mice treated with the respective companion antibiotics alone.

At the end of time points week +1, week +2, and week +4, the mean lung M. abscessus burdens in the mice treated with omadacycline in combination with cefoxitin were statistically significant compared to the mice treated with PBS alone but not significant compared to the mice treated with omadacycline plus cefoxitin except at time point week +2.

The mean lung M. abscessus burdens in the mice treated with rifabutin, amikacin, or clofazimine in combination with omadacycline versus those in the mice treated with PBS alone were statistically significant at the end of time points week +1 and week +2, but the mean lung M. abscessus burdens were not significant at the week +4 time point. In the case of mice treated with omadacycline in combination with rifabutin, amikacin, or clofazimine versus rifabutin, amikacin, or clofazimine alone, the mean lung M. abscessus burdens were statistically significant at the end of time point week +2 for omadacycline plus rifabutin versus rifabutin only.

### Determination of MSW and MPC of omadacycline.

M. abscessus isolates ATCC 19977, M9501, and M9507 were used to determine mutant selection window (MSW), mutant prevention concentration (MPC), and frequencies of spontaneous resistance and persistence to omadacycline. MSW represents the lowest and highest omadacycline concentrations above its MIC at which distinct M. abscessus colonies could be isolated. MPC represents the omadacycline concentration at or above which resistant M. abscessus could not be isolated. An axenic culture of each strain of M. abscessus was prepared by inoculating a frozen stock of each into Middlebrook 7H9 and incubating at 37°C in an orbital shaker at 220 rpm. Each culture was grown to exponential phase (*A*_600_ of ~1.0 to 1.5) and used to prepare a suspension at an *A*_600_ of 1.0 by diluting in the same broth. An 0.5-mL volume of this suspension was inoculated onto CAMH agar plates supplemented with omadacycline at 1, 2, 4, 8, 10, 16, and 20 times the MIC corresponding to the specific M. abscessus isolate and incubated at 37°C until M. abscessus growth or lack thereof was ascertained, at 7 days or after an additional 7 days to identify any additional slow-growing colonies. For M. abscessus M9501, an additional concentration of 24 times the MIC was evaluated.

### Determination of frequencies of spontaneous resistance or persistence to omadacycline.

An axenic culture of each M. abscessus strain was grown to exponential phase, and a suspension at an *A*_600_ of 1.0 was prepared as described above. An 0.5-mL volume of this suspension was inoculated onto each of 10 total CAMH agar plates supplemented with omadacycline at the median MSW concentration specific to the respective M. abscessus isolate (ATCC 19977 at 20 times the MIC [7.5 μg/mL], M9501 at 24 times the MIC [6 μg/mL], and M9507 at 14 times the MIC [7 μg/mL]) and incubated at 37°C until distinct colonies were observed, and CFU was enumerated from each plate.

The plates were evaluated at two different time points. Growth was evaluated first after 7 days of incubation, which is the standard incubation time for drug resistance experiments, and second, after 10 to 14 days of incubation, when there was insufficient growth at day 7 on CAMH agar containing omadacycline. Over time, the concentration of omadacycline can be expected to decrease due to oxidation. If the initial inoculum of M. abscessus included persisters, this subpopulation can be expected to grow when the concentration of omadacycline reaches below its MIC. Therefore, to rigorously test for the presence of a persister subpopulation and to quantify this population, we incubated the test plates for a longer duration and enumerated additional colonies that appeared at 10 and 14 days of incubation. We did not observe an additional increase in CFU after 14 days of incubation for any isolate.

To verify if colonies that appeared after 7 days were truly resistant to omadacycline and not transiently tolerant (i.e., persisters), we randomly sampled nine colonies derived from each parent strain that appeared after 7 days of incubation in omadacycline. Each isolated colony (here referred to as a “putative mutant”) (*n *= 27) was cultured in CAMHB, and the omadacycline MIC was assessed in comparison to the parent strain to verify that there was indeed a sustained increase in the MIC. The proportion of putative mutants that met this resistance criterion (resistance here defined as greater than a 4-fold increase in MIC compared to that for the parent that is sustained after passage) was considered to be omadacycline resistant. This proportion was used to determine the total number of resistant colonies arising from each parent strain in the 10 corresponding selection agar plates. The frequency of spontaneous resistance to omadacycline was determined by dividing the total number of resistant CFU by the total number of input M. abscessus CFU. The frequency of persistence to omadacycline was determined by dividing the total number of persistent CFU by the total number of input M. abscessus CFU. The input M. abscessus CFU for each strain was determined by enumerating CFU in the initial suspension on CAMH agar without selection drug as described above.

To verify if putative mutants carried a stable increase in omadacycline MIC that was heritable and therefore met the criteria for resistance to omadacycline (and not transient tolerance), three M. abscessus putative mutants derived from each parent strain with the greatest increases in omadacycline MIC were sequentially passaged in drug-free CAMHB and CAMH agar. Isolated putative mutants from MSW omadacycline plates (labeled with prefix T0) were transferred to drug-free CAMHB and inoculated on CAMH agar when the culture was grown to exponential phase (*A*_600_ of ~1.0 to 1.5). The cycle from drug-free CAMHB to CAMH agar represented one generational passage and was repeated five times. The putative mutants after five passages were labeled with the suffix -T5x. The parent strains, ATCC 19977, M9501, and M9507, were also similarly passaged to control for any nonspecific MIC shifts and are identified with the suffix -T5.

After five rounds of passage of putative mutants and their parent strains M9501 and M9507 in drug-free medium, MICs against an eight-drug panel (omadacycline, azithromycin, cefoxitin, tigecycline, clarithromycin, cefdinir, imipenem, and rifabutin) were determined, to assess for a stably elevated omadacycline MIC and any cross-resistance to comparator drugs. MIC assays were performed in duplicate, and the average MIC was calculated.
