# Supplementary material for: Efficacy of Omadacycline-Containing Regimen in a Mouse Model of Pulmonary Mycobacteroides abscessus Disease
Source: mSphere. 2023 Mar 13;8(2):e00665-22. doi: 10.1128/msphere.00665-22 (PMC10117123; doi:10.1128/msphere.00665-22)
Supplement: TABLE S1 [file msphere.00665-22-s0001.pdf]

Table S1: Statistical assessment of *Mab* burden between groups of mice receiving different treatments.

Panel of Figure 1 in the manuscript	PBS or single antibiotic versus antibiotics in combination	Week +1		Week +2		Week +4	
		p-value		*p*-value		*p*-value	
A	PBS VS OMC	0.085	ns	0.015	*	0.010	**
	PBS VS LZD	0.077	ns	0.009	**	0.283	ns
	PBS VS OMC+LZD	0.016	*	0.006	**	0.007	**
	OMC VS LZD	0.943	ns	0.431	ns	0.527	ns
	OMC VS OMC+LZD	0.308	ns	0.153	ns	0.748	ns
	LZD VS OMC+LZD	0.342	ns	0.423	ns	0.404	ns
B	PBS VS OMC	0.085	ns	0.015	*	0.010	**
	PBS VS FOX	0.005	**	0.006	**	0.000	**
	PBS VS OMC+FOX	0.000	**	0.001	**	0.000	**
	OMC VS FOX	0.537	ns	0.886	ns	0.297	ns
	OMC VS OMC+FOX	0.136	ns	0.056	ns	0.391	ns
	FOX VS OMC+FOX	0.232	ns	0.024	ns	0.908	ns
C	PBS VS OMC	0.085	ns	0.015	*	0.010	**
	PBS VS IMI	0.001	**	0.000	**	0.000	**
	PBS VS OMC+IMI	0.000	**	0.001	**	0.000	**
	OMC VS IMI	0.407	ns	0.012	*	0.055	ns
	OMC VS OMC+IMI	0.151	ns	0.073	ns	0.199	ns
	IMI VS OMC+IMI	0.308	ns	0.442	ns	0.360	ns
D	PBS VS OMC	0.085	ns	0.015	*	0.010	**
	PBS VS BIA	0.000	**	0.000	**	0.000	**
	PBS VS OMC+BIA	0.000	**	0.000	**	0.000	**
	OMC VS BIA	0.142	ns	0.005	**	0.052	ns
	OMC VS OMC+BIA	0.036	*	0.006	**	0.047	*
	BIA VS OMC+BIA	0.159	ns	0.696	ns	0.982	ns
E	PBS VS OMC	0.085	ns	0.015	**	0.010	*
	PBS VS AZM	0.221	ns	0.190	ns	0.439	ns
	PBS VS OMC+AZM	0.007	**	0.028	*	0.005	**
	OMC VS AZM	0.650	ns	0.299	ns	0.312	ns
	OMC VS OMC+AZM	0.826	ns	0.879	ns	0.915	ns
	AZM VS OMC+AZM	0.700	ns	0.385	ns	0.270	ns
F	PBS VS OMC	0.085	ns	0.015	*	0.010	**
	PBS VS RFB	0.797	ns	0.126	ns	0.507	ns
	PBS VS OMC+RFB	0.001	**	0.001	**	0.624	ns
	OMC VS RFB	0.433	ns	0.256	ns	0.287	ns
	OMC VS OMC+RFB	0.093	ns	0.092	ns	0.250	ns
	RFB VS OMC+RFB	0.062	ns	0.016	*	0.912	ns
G	PBS VS OMC	0.085	ns	0.015	*	0.010	**
	PBS VS AMK	0.000	**	0.000	**	0.062	ns
	PBS VS OMC+AMK	0.027	*	0.025	*	0.172	ns
	OMC VS AMK	0.098	ns	0.041	*	0.919	ns
	OMC VS OMC+AMK	0.762	ns	0.733	ns	0.477	ns
	AMK VS OMC+AMK	0.129	ns	0.242	ns	0.515	ns
H	PBS VS OMC	0.085	ns	0.015	*	0.010	**
	PBS VS CFZ	0.056	ns	0.077	ns	0.003	**
	PBS VS OMC+CFZ	0.018	*	0.026	*	0.141	ns
	OMC VS CFZ	0.655	ns	0.720	ns	0.637	ns
	OMC VS OMC+CFZ	0.588	ns	0.862	ns	0.177	ns
	CFZ VS OMC+CFZ	0.984	ns	0.650	ns	0.077	ns
I	PBS VS OMC	0.085	ns	0.015	*	0.010	**
	PBS VS BDQ	0.324	ns	0.073	ns	0.037	*
	PBS VS OMC+BDQ	0.032	*	0.036	*	0.006	**
	OMC VS BDQ	0.796	ns	0.772	ns	0.263	ns
	OMC VS OMC+BDQ	0.639	ns	0.253	ns	0.662	ns
	BDQ VS OMC+BDQ	0.534	ns	0.645	ns	0.396	ns
