# Supplementary material for: Efficacy of Omadacycline-Containing Regimen in a Mouse Model of Pulmonary Mycobacteroides abscessus Disease
Source: mSphere. 2023 Mar 13;8(2):e00665-22. doi: 10.1128/msphere.00665-22 (PMC10117123; doi:10.1128/msphere.00665-22)
Supplement: TABLE S2 [file msphere.00665-22-s0002.pdf]

Table S2. *M. abscessus* isolates selected for generational passaging with omadacycline MIC shifts at T0 and T5 generations

**Parent *M. abscessus* ID**	**omadacycline MIC of parent (µg/ml)**	***M. abscessus* mutant ID**	**omadacycline MIC vs. T0 mutants (µg/ml)**	**omadacycline MIC fold change in T0 isolates**	**omadacycline MIC vs. parent strain after passage (µg/ml)**	**omadacycline MIC vs. T5 mutants after passage (µg/ml)**	**Fold change in omadacycline MIC of T5 compared to T0**
M9501	0.375	M9501-T52	4	10.7	0.75	3	4
M9501	0.375	M9501-T56	2	5.3	0.75	2	2.7
M9501	0.375	M9501-T57	2	5.3	0.75	1.5	2
M9507	0.5	M9507-T51	5	10	0.75	6	8
