# Supplementary material for: Efficacy of Omadacycline-Containing Regimen in a Mouse Model of Pulmonary Mycobacteroides abscessus Disease
Source: mSphere. 2023 Mar 13;8(2):e00665-22. doi: 10.1128/msphere.00665-22 (PMC10117123; doi:10.1128/msphere.00665-22)
Supplement: TABLE S3 [file msphere.00665-22-s0003.pdf]

Table S3. MIC (µg/mL) of antibiotics commonly used to treat *M. abscessus* disease panel vs the fifth generation (T5) mutants and respective antibiotic MIC shifts

Drug	M9501 Parent	M9501-T5	M9501-T52	Fold change	M9501-T56	Fold change	M9501-T57	Fold change
**OMC**	0.375	0.75	3	4	2	2.7	1.5	2
**AZM**	0.5	0.5	0.5	1	0.5	1	0.75	1.5
**FOX**	32	16	16	1	16	1	16	1
**TGC**	0.25	0.75	1.5	2	0.5	0.67	0.75	1
**CLR**	<0.06	0.063	0.094	1.5	0.094	1.5	0.063	1
**CDR**	64	32	12	0.38	24	0.75	32	1
**IMI**	16	40	64	1.6	48	1.2	24	0.6
**RFB**	8	4	3	0.75	3	0.75	4	1

Drug	M9507 Parent	M9507-T5	M9507-T51	Fold change
**OMC**	0.5	0.75	6	**8**
**AZM**	32	12	8	0.67
**FOX**	32	12	16	1.3
**TGC**	0.25	0.75	2	2.7
**CLR**	4	0.88	2.5	2.86
**CDR**	96	80	128	1.6
**IMI**	24	40	32	0.8
**RFB**	8	6	6	1
